# Obesity and cancer: unravelling the microbiome’s hidden role

**DOI:** 10.3389/fnut.2025.1602603

**Published:** 2025-06-09

**Authors:** Christine Gaskell, Ross MacDonald, Eiman Aleem, Ghizlane Bendriss

**Affiliations:** ^1^Weill Cornell Medicine Qatar, Doha, Qatar; ^2^Cancer, Infection and Therapeutics Research Group, School of Applied and Health Sciences, College of Health and Life Sciences, London South Bank University, London, United Kingdom

**Keywords:** bidirectional, cancer, gut microbiota, lifestyle, obesity, physical activity

## Abstract

Obesity has been implicated as the driving force of many diseases including cancer through multiple biological mechanisms, including gut microbial imbalances, compromised intestinal barrier integrity, persistent low-grade inflammation, and alterations in energy uptake. As lifestyle factors such as diet, physical activity, and sleep are known to influence disease susceptibility, understanding the role of the gut microbiome in these interactions is critical. A deeper understanding of the intricate connections between gut microbiota, obesity, and various cancers could be used to better inform effective strategies for disease prevention and treatment. Investigating the microbiome’s influence on tumor progression and systemic metabolic health may be the way forward for novel diagnostic and therapeutic approaches. It is essential to investigate how lifestyle factors are linked to both obesity and cancer, and what role the microbiome is playing. This review synthesizes current research on the mechanistic role of the gut microbiome in obesity and cancer, highlighting its potential role in early detection, prognosis, and its use as a targeted intervention to restore gut eubiosis.

## 1 Introduction

Obesity, as defined as those with a BMI > 30 represents a complex chronic disease that is one of the most important public health challenges of the 21st century. The WHO (World Health Organization) reported that obesity rates have seen a vast growth, almost tripling since 1975, with over 650 million adults classified as obese in 2022 (1). Characterized by excessive adipose tissue accumulation, the health significance of obesity proceeds beyond the immediate and obvious impact on body weight, physical immobility and skeletal issues that comes with carrying an excess of weight. It serves as a risk factor for several complex health conditions, one of which is cancer. Obesity is known to induce chronic, low-level inflammation in the body which creates a pro-tumour environment. Obesity is linked to numerous types of cancers including breast, colorectal, esophageal, kidney, gallbladder, uterine, pancreatic, and liver (2).

In 2022 there were approximately 20 million new cases of cancer diagnosed globally and 9.7 million deaths from cancer (3) Female breast cancer is now the most diagnosed cancer, closely followed by lung, colorectal (CRC) and prostate cancer (4). Cancer represents a complex disease characterized by the uncontrolled growth of abnormal cells that have the ability to change their surrounding environment to make it more favorable, destroy surrounding tissues and spread to distant parts of the body through metastasis. At the most basic level, cancer is caused by changes to the genes that disrupt normal cellular mechanisms of growth, division, and apoptosis. This change is often brought about by environmental determinants and lifestyle factors including obesity.

The human gut microbiome is a complex ecosystem composed of trillions of microorganisms, including bacteria, viruses, fungi, and protozoa, that reside in the gastrointestinal tract. This microbial community contains over 100 trillion cells and plays a far bigger role than previously thought. The gut microbiome is now known to influence host metabolism, immune function, neurological processes, and overall health (5). Scientific advances have revealed that the gut microbiome plays an active part in a number of physiological processes, with the composition and diversity linked to a wide range of health conditions, from metabolic disorders and inflammatory diseases to cancer and neurological pathologies (6, 7). This review examines the nexus between lifestyle factors – obesity – cancer and proposes a mechanism involving gut microbes to explain how lifestyle can relate to obesity and cancer. Mechanistic relationships between the gut microbiome and both obesity and cancer, with particular emphasis on how lifestyle factors, specifically diet, physical activity, and sleep patterns influence microbial communities. While previous reviews have explored these topics separately, our analysis uniquely integrates these interconnected elements to address a critical gap in the literature: the comprehensive understanding of how lifestyle modifications alter the gut microbiome to potentially mitigate disease progression.

## 2 Methods

Before the initial search, key terms and concepts were decided to ensure a concise and accurate search of the available literature. Keywords were chosen based on our search questions: 1/ causes of obesity; 2/ link between diet and microbiome; 3/ link between gut microbes, metabolites and cancer/obesity/epigenetics; 4/ association between obesity and physical activity; 5/ gut metabolites and sleep/hunger/psychiatric disorders; 6/ link between physical activity/sleep/psychiatric disorders and diet.; 7/ probiotics and fecal transplant and microbiome. Using the pre-decided key terms and Boolean operators PubMed was searched to identify relevant studies. Specific filters were applied to narrow the results (e.g., publication date, language). A manual search of the reference lists from key articles was performed to identify additional studies not captured in the initial database search.

## 3 Obesity and cancer

Obesity is one of the most significant modifiable risk factors for cancer development, second only to tobacco use in preventable cancer causes. Excess adipose tissue increases the risk for at least 13 different types of cancers, including endometrial, esophageal adenocarcinoma, colorectal, postmenopausal breast, prostate, and renal cell cancers (2, 8). The biological mechanisms underlying this relationship are multifaceted. As global obesity rates continue to rise, understanding these connections has become increasingly urgent.

The microbiome was reported to play an important role in some cancers including CRC. CRC is the most common cancer for both sexes in the US and the third most prevalent cancer worldwide: previously a cancer of high economic countries, an increase in cases from low-income countries is thought to be due to the rapid change in diet from traditional foods to one that more closely resembles the Western Diet (WD) (9, 10). The sharp rise in CRC, particularly early-onset CRC, is linked to obesity and a diet high in saturated fats and low in fibre (11, 12). Multiple studies have highlighted the change in bacterial species in cases of CRC as compared to healthy individuals, with the most noted differences being a loss of diversity, and the presence in the colon of bacteria normally associated with the oral cavity in healthy individuals, notably genotoxin-producing *Fusobacterium nucleatum* (13). Indeed, it has even been suggested (14) that the gut microbiota could be used as a tool to identify colonic lesions, as CRC patients contained higher levels of bacterial taxa traditionally thought of as oral pathogens, notably *Fusobacterium*, *Porphyromonas*, *Peptostreptococus*, *Gemella*, *Parvimonas*, and *Prevotella*. This increase in pathogenic bacterial species and decrease in butyrate-producing bacteria leads to a change in the local immune response and a shift in mucosal state to one that supports tumor progression (12). The relationship between dysbiosis of the gut microbiome and cancer is best studied for CRC (15, 16), but there is some evidence for the role of the microbiome in other cancers. Breast cancer is the most common cancer of women worldwide. It has been shown that women with breast cancer have a different gut microbiota than women without breast cancer (17).

*Fusobacterium nucleatum* has also been implicated in promoting tumor progression in pancreatic ductal adenocarcinoma, the most prevalent form of pancreatic cancer through triggering chronic inflammation and the increased release of cytokines (18, 19). Additionally, gut microbiota-derived bile acids have been shown to suppress immune surveillance, and antibiotic intervention in mouse models of liver cancer has demonstrated the potential to inhibit tumor growth. The lung microbiome, influenced by microbial communities from the oral, nasal, and gastrointestinal tracts, has also emerged as a contributing factor in lung cancer (20–22). Notably, individuals with lung cancer often exhibit reduced microbial diversity within lung tissue (23).

## 4 Factors affecting the gut microbiome

**Figure 1:**
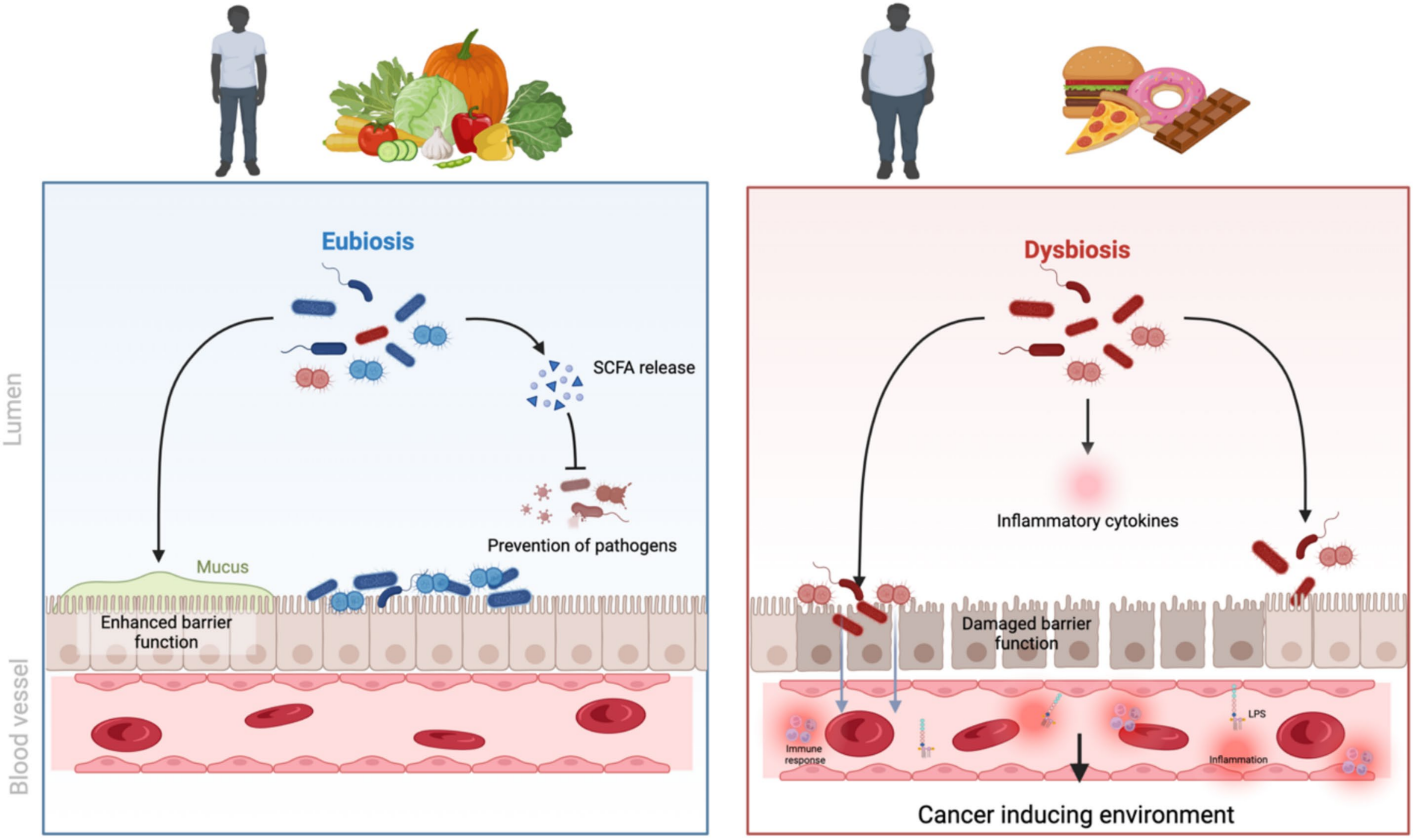
The effect of an unhealthy diet and obesity on gut microbiome and cancer initiation. Created in BioRender.

The composition of diet plays a role in obesity. Highly processed carbohydrates can cause rapid glucose absorption, triggering insulin spikes and promoting adipose storage; added sugars are associated with visceral adiposity; and a reduced fibre intake can lead to constipation and affect gut microbiome health (5). Excessive consumption of saturated and trans fats is linked to inflammation and metabolic dysregulation (24), while the quality of protein consumed in a diet can affect satiety, muscle protein synthesis, and body composition (25). One dietary factor now recognized as playing a major role is the high consumption of ultra-processed foods that are designed to be hyperpalatable but have a poor nutrient profile (Figure 1). Lane, Gamage (26) found that energy from ultra-processed foods ranges from between 42 to 58% in countries such as Australia and the US. Other factors that play a role in diet and obesity are hormonal regulation, chrononutrition, physical activity levels, and sleep (27). The quality of the food consumed can impact the signalling of the hunger and satiety hormones leptin and ghrelin, as well as the stress hormone cortisol (28). Irregular eating patterns, fasting and late-night snacking have also been associated with an increased risk of obesity due to the effect of meal timing on cell cycle regulation (29).

While diet is a major contributing factor in obesity, it is crucial to recognize that other environmental factors, such as antibiotic exposure, medications, and pollutants, also play significant roles. Accumulating evidence suggests that these factors may contribute to the development of obesity by influencing the gut microbiota (7). Early-life antibiotic exposure has been associated with an increased risk of obesity due to its effects on the intestinal microbial community (30). This may be due to dramatic changes in the intestinal microbiota in response to oral antibiotic treatments: a study of Finnish children (31) found those who had consumed antibiotics belonging to the macrolide group had a long-lasting shift in the microbial composition that included a depletion of Actinobacteria and increases in Bacteroidetes and Proteobacteria. Similarly, environmental pollutants and certain medications can disrupt the delicate balance of the gut microbiome, promoting dysbiosis and potentially impacting energy metabolism and weight regulation (32). The role of hormones in obesity has also gathered interest recently, due to the changing environment and the inclusion of Endocrine Disrupting Chemicals (EDC) in everyday products such as food packaging. These EDC are thought to alter lipid metabolism and alter the hormonal pathway for satiety leading to weight gain and alteration in the gut microbiome (33, 34).

Studies have shown that the gut microbiomes of lean and obese individuals differ significantly in their composition (6, 35–37). Obese individuals tend to have a higher abundance of Firmicutes and a lower abundance of Bacteroidetes, a microbial profile that has been associated with increased energy harvesting and storage (38). This shift in the gut microbiome composition is thought to be driven, in part, by the WD, which is typically high in fat and low in fibre. While earlier research suggested a consistent pattern of higher Firmicutes and lower Bacteroidetes in obese individuals, more recent studies reveal a more complex picture (39). The ratio of Firmicutes to Bacteroidetes may not be as consistent as initially thought, and specific changes at the species level seem more relevant. Alterations to the ratio of Firmicutes to Bacteroidetes is affected by diet and weight loss can impact the diversity found in an individual’s gut. Obese individuals often exhibit reduced microbial diversity and an altered abundance of specific bacterial groups (40). For instance, some studies have reported a depletion of *Blautia* species in obese children, which correlates with intestinal inflammation and worsened metabolic phenotype (41). *Akkermansia muciniphila*, a bacterium known for its mucin-degrading properties, has gained attention for its potential role in modulating metabolism and improving gut health (42). In addition, although most studies focus on bacteria, some studies also suggest obesity changes the human gut mycobiome (43). It’s important to consider that the relationship between specific microbial changes and obesity can be influenced by various factors, including diet, genetics, and even geographical location (44).

## 5 The microbiome -metabolome-obesity-cancer link

The gut microbiome’s influence goes beyond that of energy homeostasis, shaping the host’s metabolic landscape through interactions with various physiological pathways, influencing cancer risk. Obesity-related shifts in the gut microbiome led to altered production of key metabolites, including short-chain fatty acids (SCFAs), which play a crucial role in modulating inflammation, immune responses, and metabolic regulation. These microbial changes influence cytokine production, promoting a pro-inflammatory environment that can contribute to tumorigenesis (Table 1). Additionally, disruptions in gut-derived hormones and neurotransmitters affect metabolic signaling, further exacerbating obesity and increasing cancer susceptibility. Epigenetic modifications induced by microbial metabolites further regulate gene expression, reinforcing the complex interplay between the gut microbiome, obesity, and cancer.

**Table 1:** Species and phylum of bacteria and its relation to obesity and cancer.

Gut bacteria species	Phylum	Effect in relation to obesity	Effect in relation to cancer
*Bacteroides fragilis*	Bacteroidetes	Increase in IL-10 production in women increasing several pro-inflammatory cytokines (38)	Increase in IL-17 levels which has Pro angiogenesis effects (45)
*Akkermansia muciniphilia*	Verrucomicrobia	Regulates metabolism and energy hemostasis and improves insulin sensitivity (46)	Degrades mucin as a main source of carbon and contributes to increasing intestinal barrier integrity (47)
*Escherichia Coli*	Proteobacteria	Increases energy harvest and impairs glucose homeostasis (48)	Promotes intestinal inflammation (45)
*Bifidobacterium adolescentis*	Actinobacteria	Decreases visceral fat accumulation and increases insulin sensitivity (49)	Alleviates the symptoms of cancer and maintains gut wall integrity (50)
*Faecalibacterium prausnitzil*	Firmicutes	Modulates systemic inflammation and enhances gut barrier integrity (51)	Therapeutic effect in induced colon inflammation (52)
*Staphylococcus aureus*	Firmicutes	Creates low grade inflammation (38)	Tumour promoting, causes DNA damage and disrupts signaling pathways (53)
*Fusobacterium nucleatum*	Fusobacteria	Pro inflammatory properties (54)	Promotion of tumour microenvironment through inhibition of NK or T cells (45)

### 5.1 Short chain fatty acids

A number of metabolomic changes observed in obesity have been directly linked to the gut microbiome composition. Microbial fermentation of dietary fibre found in foods such as vegetables produce short-chain fatty acids (SCFAs) such as acetate, propionate, and butyrate (55). In particular, Bacteroidetes in the Firmicutes are responsible for producing butyrate and propionate. Firmicutes have both a harmful effect, through pro-inflammatory response and a beneficial response depending on the bacteria species. Butyrate in particular may play a number of roles in reducing inflammation or reducing cancer risk. It serves as the main energy source for the cells of the colon (56) and plays a crucial role in maintaining intestinal barrier integrity by facilitating tight junction assembly (57) as well as being an anti-inflammatory agent, inhibiting pro-inflammatory cytokines, and supporting regulatory T-cell function (58). Butyrate produced by gut bacteria also regulates gut mucus barrier repair, potentially by polarizing macrophages into a M2 state (59). SCFAs also act to down-regulate fatty acids synthesis and lipolysis, leading to a decrease in body weight and reduced likelihood of obesity (56, 60). Butyrate has been observed *in vitro* to inhibit proliferation and promote apoptosis in some cancer cell lines (61).

### 5.2 Cytokines

A dysbiosis in the gut microbiome as the result of obesity leads to a change in metabolites such as SCFA that cause a pro-inflammatory state characterized by increased levels of pro-inflammatory cytokines such as IL-1, IL-6, and TNF-*α*, the so-called “inflammatory triad” (62). This systemic inflammatory state may be initiated or enhanced through multiple adipose tissue-dependent mechanisms. Adipose tissue, particularly in the context of obesity, undergoes pathological expansion characterized by adipocyte hypertrophy, hypoxia, and stress responses that trigger the production of pro-inflammatory cytokines including TNF-*α*, IL-6, and IL-1β. These mechanisms are described further in the following sections (62, 63).

There are two forms of adipose tissue in the human body, white adipose tissue (WAT) and brown adipose tissue (BAT) (64). It is now known that WAT is an active endocrine organ, responsible for the production and secretion of various adipokines, with leptin and adiponectin being among the most physiologically significant. Leptin regulates appetite and energy balance by influencing the hypothalamus, while adiponectin is responsible for enhancing insulin sensitivity and is known to have anti-inflammatory properties, with lower levels associated with obesity, diabetes, and increased cancer risk (65–67). In contrast, resistin, primarily produced by circulating monocytes in human adipose tissue, plays a significant role in metabolic dysfunction by impairing insulin signaling and promoting systemic inflammation. By interfering with insulin action, resistin contributes to the development of insulin resistance, a key factor in metabolic disorders such as type 2 diabetes, and cancers such as breast, colorectal, pancreatic and endometrial (68–70). Additionally, it enhances the release of pro-inflammatory cytokines from mononuclear cells, further exacerbating chronic low-grade inflammation. This inflammatory response not only disrupts glucose homeostasis but also contributes to the progression of obesity-related complications, cardiovascular diseases, certain cancers and other metabolic syndromes. Visfatin is also involved in glucose metabolism and immune response, functioning both as a cytokine and an adipokine (67). Visfatin exhibits insulin-like properties by binding to insulin receptors at a site distinct from insulin itself, thereby promoting glucose uptake in peripheral tissues. Furthermore, visfatin plays a crucial role in inflammation, with elevated levels observed in various inflammatory conditions and obesity (65). Its expression increases in response to proinflammatory cytokines, and it subsequently stimulates the production of inflammatory mediators such as IL-6, TNF-*α*, and IL-1β, creating a potential feedback loop that may exacerbate chronic low-grade inflammation (65, 71, 72).

Whilst both lean and obese individuals have WAT present, the quantity and composition differ with obese individuals having a dysregulated configuration leading to an increased pro-inflammatory state and a reduction in adiponectin (64). Adiponectin, a 247-amino acid peptide has been shown in multiple studies to enhance insulin sensitivity in muscle and adipocytes, it improves endothelial health through local nitric oxide production and promotes weight loss through increased oxidation of lipids. Adiponectin’s ability to suppress tumor growth and angiogenesis while triggering apoptosis indicates that lower circulating levels of adiponectin as found in obese individuals could be a mechanism linking excess weight to tumor development (65).

**Figure 2:**
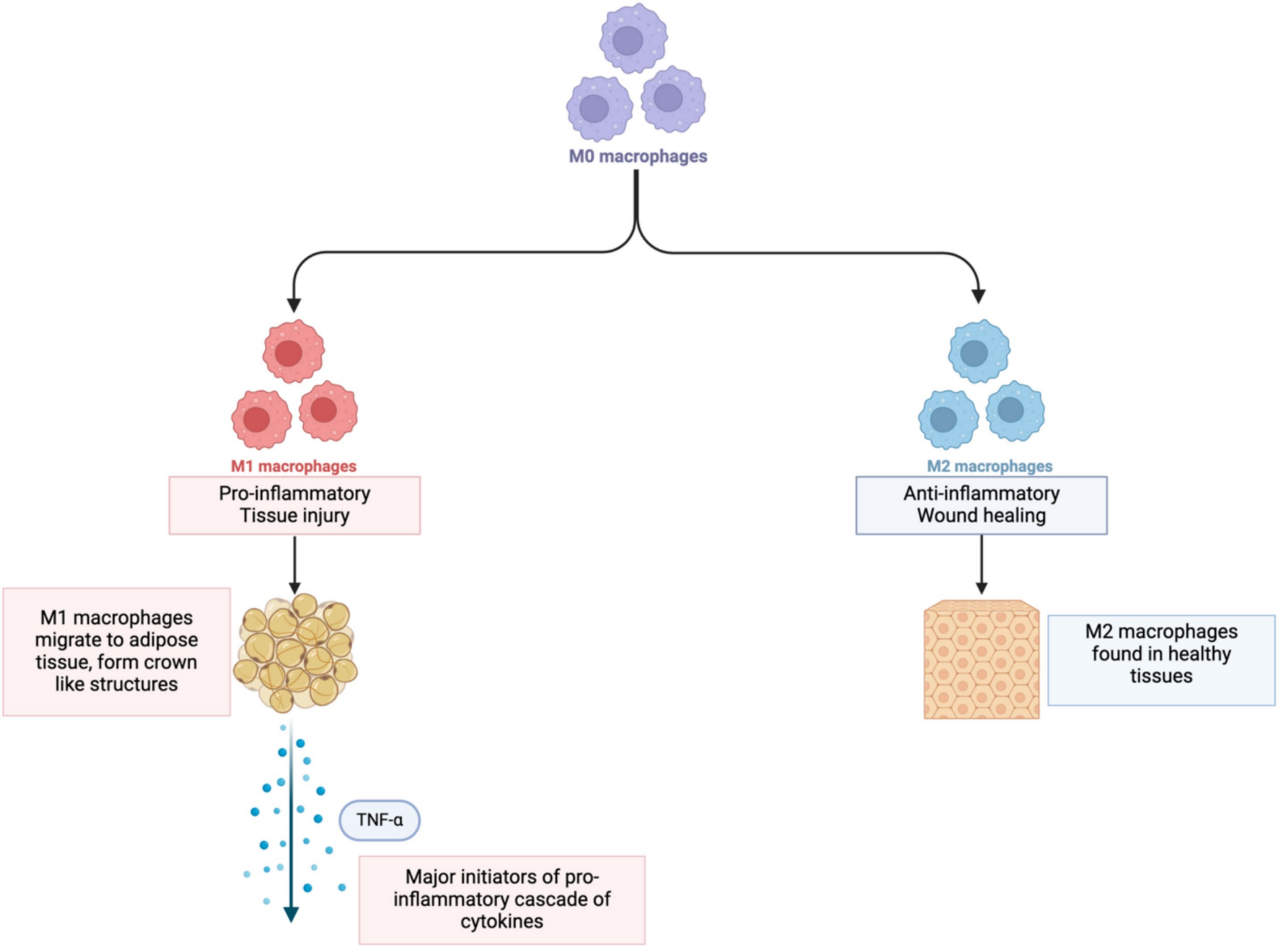
Change in monocytes to M1 and M2 macrophages. Created in BioRender.

Within the WAT, fat-storing adipocytes are responsible for a host of responses within the body such as metabolism of lipids and glucose, inflammation, blood pressure and angiogenesis (67). An increase in body fat that causes an increase in the volume of the adipocyte is referred to as hypertrophy (73); hypertrophy can lead to adipocyte hypoxia when blood vessels do not grow quickly enough to match the expansion of the adipocytes. This leads to a low-oxygen environment causing the cells to produce cytokines and chemokines that attract circulating monocytes (Figure 2).

Upon infiltration of the hypoxic adipose tissue, M1 macrophages become major initiators of a pro-inflammatory cascade of cytokines, particularly TNF-*α*, which is that is responsible for disrupting insulin signaling, reducing adiponectin, and increasing free fatty acids. It also stimulates other inflammatory molecules (74) which work in parallel with TNF-*α* to promote inflammation by affecting insulin sensitivity, fat metabolism, and appetite regulation, and deregulating expression of genes involved in tumor growth via JAK/STAT pathways (64).

While TNF-α is produced as a result of tissue stress in WAT, it has also been reported that one third of total circulating IL-6 originates from adipose tissue (73). Maqoud, Calabrese (75) also found that in individuals placed into three groups (group 1 – overweight; group 2 – obese; group 3 – morbidly obese) there was a significant increase in IL-6 levels between group 1 and group 3 (but no change in levels between the groups for TNF-*α*).

Together, increasing levels of these cytokines create a harmful feedback loop, with TNF-α and IL-6 recruiting more M1 macrophages, whilst also causing adipocyte dysregulation. The malfunctioning adipocytes produce more inflammatory signals that attract even more M1 macrophages, and the cycle continues.

A study by Xue et al. (76) investigated the association between gut microbiota and inflammatory cytokines, finding seven significant associations between bacteria phyla such as *Euryarchaeota* with IL-2 and IL-8. This is of particular interest in the case of gastric cancer in which *Helicobacter pylori* is known to induce the production of IL-8, driving inflammation with the potential to lead to cancer (77).

### 5.3 Hormones

Gut dysbiosis can directly affect hormone production and signaling, including that of hormones involved in appetite regulation, satiety, and stress response. Altered gut hormone levels can lead to increased food intake, cravings for unhealthy foods, and decreased energy expenditure, contributing to weight gain and obesity.

The relationship between dysbiosis of the gut microbiome and cancer is best studied for CRC (15, 16), but there is some evidence for the role of the microbiome in other cancers. Breast cancer is the most common cancer of women worldwide. It has been shown that women with breast cancer have a different gut microbiota than women without breast cancer (17).

As highlighted in the previous section, adipose tissue is an active endocrine organ that influences hormone metabolism. Excess adipose tissue is a core attribute of obesity and can significantly affect the levels and balance of various sex hormones, including estrogen, androgens, and progesterone, all of which play critical roles in the development and progression of hormone-sensitive cancers such as breast, endometrial, and prostate cancer. Additionally, adipose tissue is a significant source of aromatase, an enzyme that catalyzes the conversion of androgens into estrogens, further contributing to the elevated estrogen levels associated with obesity (78). This positive relationship between fat mass, aromatase expression, and higher estrogen levels is particularly prominent in the post-menopausal state (79). The complex interplay between obesity, metabolic dysregulation, and altered sex hormone levels creates an inflammatory state that can promote a tumorigenic environment, thereby increasing the risk of hormone-sensitive cancers (80–83). If gut microbiome alterations play a role in breast cancer, the mechanism is still not fully understood, and Baker, Al-Nakkash (84) note that estrogen receptor-positive (ER+) breast cancer has been linked to a hyperactive estrobolome – a collection of genes in commensal bacteria responsible for estrogen metabolism that causes increased intestinal absorption of free estrogen leading to an elevated risk of developing breast cancer. The gut microbiota is also responsible for modulating the estrogen-metabolizing enzyme beta-glucuronidase which influences estrogen metabolism (85).

The discussion around obesity and breast cancer in women considers two distinct groups: postmenopausal and premenopausal. This is due to the differences in circulating hormone profiles in the different stages of a woman’s life (86). In postmenopausal women, an increased BMI is linked to an increased incidence of breast cancer; however, in premenopausal women, obesity has been shown to have the opposite effect and play a protective role (82, 87, 88). Adipose tissue produces excess estrogen, which is implicated in hormone-sensitive cancers such as breast and endometrial cancer. Adipose tissue becomes the main source of estrogen production in postmenopausal women with obesity. This is due to androgens that are released from the adrenal glands being converted into estrogens by adipocytes, in a process called aromatization. As a result, the greater the levels of adipose tissue, the more androgens are converted, and the greater the circulating levels of estrogen. Adipose tissue also secretes cytokines, growth factors, and inflammatory molecules that further impact aromatization, leading to increased estrogen levels. Elevated estrogen levels interact with hormone-sensitive breast tissue, increasing the risk of estrogen receptor-positive (ER+) breast cancer (65) via an increased cell proliferation and reduction in apoptosis (89). In contrast, estrogen production in premenopausal women occurs mostly in the ovaries: this results in obesity having a lesser impact on estrogen production as estrogen levels in premenopausal women tend to be lower because of significant absorption of estradiol into fat tissue and an increased rate of estrogen metabolism and clearance by the liver (86). Loh et al. (90) found that obesity in premenopausal women had a protective effect for ER + breast cancer. Choi, Choi (87) also found that obesity and/or higher waist circumference was linked to an increased incidence of gastric cancer in postmenopausal women, although no association was found between obesity and or waist circumference and gastric cancer in premenopausal women. Changes to the microbiome diversity involved in estrogen and hormone metabolism can also lead to an increased level of circulating estrogen, increasing the risk of breast cancer. Additionally, a decreased abundance of *Methylobacterium* in breast cancer patients has been associated with more invasive tumors (91).

In the case of endometrial cancer, there are interactions between hormonal fluctuations, disturbances in gut microbial balance, and chronic inflammation. Post-menopausal women exhibit a heightened cancer risk, potentially linked to shifts in gut microbial composition and intestinal environment that accompany hormonal changes (92). The menstrual cycle also plays a role in the cyclical timing of the proliferation on the endometrium. Estrogen increases cell proliferation as a normal stage in the cycle and is counter-balanced by progesterone (81). Obese women have a lower circulating level of progesterone which leads to continued cell proliferation. Once menopause has occurred, ovarian estrogen production starts to decline rapidly and is replaced as previously described by production in adipose tissue (83). Sex Hormone-Binding Globulin (SHBG) levels are lowered in those with obesity, meaning more estrogen and testosterone are circulating. Lowering of SHBG occurs due to hyperinsulinemia increasing insulin growth factor-1 (IGF-1) levels, creating a potentially cancer inducing environment (78, 93). There are alterations in the diversity and structure of the microbial community in endometrial cancer compared to healthy controls. Patients with endometrial cancer had a reduction in alpha diversity, with a shift from anti-inflammatory Firmicutes and Clostridia to a pro-inflammatory Proteobacteria-dominated microbiome (94). This dysbiosis appears connected to estrogen metabolism disruption, suggesting the gut microbiome as a critical mediator of endometrial cancer risk (95).

Geographical variations in endometrial cancer show that Europe and North America have the highest rates which may be due to lifestyle factors that impact gut microbial health. Unhealthy dietary patterns, sedentary behaviors, and extensive antibiotic use contribute to microbial imbalances (95). The microbiome’s role extends beyond passive observation, actively participating in hormonal regulation, inflammation modulation, and potentially cancer progression.

Prostate cancer is linked to an imbalance between estrogen and androgens. As men age their testosterone levels decline but estrogen levels normally increase (96): this increase is brought about by an increase in the activity of aromatase, which converts testosterone to estrogen. The subsequent shift in hormone levels can lead to inflammation, cell proliferation and a decrease in apoptosis causing a tumor-creating environment. However, research findings are contradictory regarding the association of obesity and prostate cancer. Several studies have found that BMI did not have a significant effect on prostate cancer total risk (90, 97–99). Although it has been noted by Agalliu et al. (97) that cases with aggressive prostate cancer had a lower BMI, they postulated it to be a result of weight loss due to the effects of the cancer. In contrast, a randomized trial by Chau, Till (98) found that increased BMI was associated with high-grade prostate cancer. Vidal, Oyekunle (100) also found obesity to be linked to high-grade prostate cancer, and those with obesity to be at a younger age for surgical treatment of prostate cancer. An explanation for the differences observed in the studies is the availability of prostate cancer screening: Agalliu et al. (97) involved participants who had received a positive histological diagnosis of prostate cancer up to 6 months before the date of enrollment, whereas participants in Hurwitz, Dogbe (99) were required to undergo screening for prostate cancer every six years, influencing detection rates, with earlier detection having a better prognosis. A pilot study by Golombos, Ayangbesan (101) investigated the gut microbiome of 20 men with high-risk prostate cancer, the study revealed a higher abundance of *Bacteroides massiliensis* in prostate cancer patients compared to the controls and a higher level of *Faecalibacterium prausnitzii and Eubacterium rectalie* in the control group.

### 5.4 Neurotransmitters

The gut microbiome acts as a neurochemical factory, directly producing or modulating the production of key neurotransmitters responsible for appetite and mood (102). These include serotonin, gamma-aminobutyric acid (GABA), dopamine and norepinephrine. Almost all of the body’s serotonin is produced within the gut (103), and it acts on the hypothalamus to regulate hunger and satiety, with deficiencies in serotonin being linked to cravings for high calorie foods and overeating (104). Specific spore-forming bacteria found in both mouse and human gut microbiota stimulate serotonin production from enterochromaffin cells (ECs) in the colon, influencing the gut mucosa and the platelets in the circulating blood effect gastrointestinal motility as well as platelet function (105). This, plus the further influence of serotonin on sleep (see Section 5.3), highlights the fact that the effects of the gut microbiome are not only local but systemic.

An imbalance in GABA, which is an inhibitory neurotransmitter, has been linked to a number of neurological disorders that include stress, anxiety, and Parkinsons disease. A number of gastrointestinal bacteria such as *Bifidobacterium, Lactobacillus, and Bacteroides* have the genetic code for glutamic acid decarboxylase (GAD), the enzyme responsible for synthesizing (GABA). In fact, *Bacteroides* are thought to be the driving force in the influence of gut microbiota on mental health by regulating GABA production (106). GABA helps regulate dopamine and serotonin, and disruptions in its production may lead to increased cravings for high-calorie foods, further promoting weight gain (107). Ma, Yan (108) tested white-to-beige adipocyte conversion using GABA supplementation as a potential treatment for obesity. They found GABA supplementation successful in not only reducing body weight but also adipose inflammation. Analysis of gut microbiota composition revealed that GABA supplementation increased beneficial bacteria such as Bacteroidetes and Akkermansia, while decreasing the levels of Firmicutes levels that are linked to obesity and inflammation. This highlights a potential gut-brain axis mechanism in obesity management.

In individuals with obesity, dopamine signaling is often dysregulated, leading to reduced dopamine receptor availability (especially D2 receptors) in the brain. This can result in overeating to compensate for reduced reward sensitivity, similar to addiction mechanisms. Additionally, dopamine influences energy expenditure and physical activity, with lower dopamine levels being linked to reduced motivation for exercise (109). Norepinephrine’s role in obesity is through the regulation of metabolism, appetite, and energy expenditure. It influences the sympathetic nervous system, which controls thermogenesis and lipolysis (110). High norepinephrine activity stimulates BAT to burn calories, promoting weight loss. Norepinephrine also affects appetite regulation by acting on the hypothalamus, contributing to increased hunger and food intake (111). Chronic stress has been known to elevate norepinephrine levels, perhaps leading to stress-induced overeating and weight gain (112).

The dysbiosis brought about by obesity disrupts not only the production of these neurotransmitters but also their regulation due to the reduction of the diversity in the gut microbiome. Obesity-associated neurotransmitter dysfunction has the potential to lead to metabolic disturbances that create a cancer inducing environment, that may act in concert with the effect of altered hormone levels (see Section 4.3). A neurotransmitter imbalance can directly influence cancer cell behavior as seen in the imbalance of serotonin which stimulates cell proliferation in colorectal and breast cancers (113). An imbalance in norepinephrine leads to activation of *β*-adrenergic signaling promoting tumor growth as well as inducing anti-apoptotic activity (114).

Emerging data suggest that cancer cells take advantage of the neurotransmitters-initiated signaling pathway to activate uncontrolled proliferation and dissemination. The gut microbiome influences neurotransmitter levels, including serotonin, dopamine, gamma-aminobutyric acid (GABA), and norepinephrine, which not only regulate mood and cognition but also affect immune function, inflammation, and tumor growth (115). Norepinephrine and dopamine have been linked to stress-related tumor growth, with chronic stress activating beta-adrenergic receptors, leading to increased inflammation, immune suppression, and enhanced tumor cell survival (116). Gut dysbiosis may alter the balance of these neurotransmitters, creating a tumor-promoting microenvironment, particularly in cancers such as colorectal, breast, and prostate cancer (117).

### 5.5 Epigenetics, the tumor microenvironment and the microbiome

Metabolomic alterations can impact gene expression and contribute to both obesity and cancer through several mechanisms which involve either changing the availability of molecules used for DNA modifications or directly blocking the function of enzymes that regulate these genetic control mechanisms (118). Gut microbial metabolites influence epigenetic modifications such as DNA methylation, by silencing tumor suppressor genes, activating oncogenic pathways, and/or modifying gene expression related to metabolism. Bacterial-derived metabolites can also cause alterations to histone acetylation and methylation, creating changes in the chromatin structure that impacts gene expression. Additionally, the modulation of non-coding RNA populations can influence gene regulation and cellular signaling pathways (119).

The altered metabolic environment in obesity can influence the tumor microenvironment, promoting cancer development and progression (120). This is seen in the elevated levels of free fatty acids that activate inflammatory pathways and increase cancer risk (121). Adipokines, originating from body fat and within the tumor capsule, can exert both proinflammatory and anti-inflammatory effects, impacting tumor growth (122). The tumor microenvironment has been compared to that of a wound healing site, with the production of proinflammatory molecules and growth factors. It is also affected by the gut microbiota through the previously highlighted epigenetic modifications (120).

### 5.6 Microbial influence of tumors

The relationship between microbiota and cancer goes beyond the intestinal environment, with studies showing associations between microbial dysbiosis and the onset and progression of multiple cancer types such as pancreatic, prostate, endometrial, and brain cancers (123–126).

There are two routes of microbial influence on tumors. The first is microbial presence at the tumor site, where specific bacteria interact with tumor and immune cells directly within the tumor microenvironment. For example, *Fusobacterium nucleatum*, commonly found in colorectal tumors and increasingly identified in pancreatic cancer tissue, has been shown to promote tumor growth and resistance to therapy through enhancement of inflammatory signaling (127, 128).

The second route involves the gut-derived microbial metabolites short-chain fatty acids, bile acids, and lipopolysaccharides. These metabolites circulate through the bloodstream and lymphatics, affecting the immune responses, influencing systemic inflammation, and endocrine pathways. In prostate and endometrial cancers, metabolites are thought to contribute to a pro-tumorigenic systemic environment by impacting hormone regulation and immune function (123, 129).

The microbial influence is highlighted in glioblastoma multiforme (GBM), where the gut-brain axis has emerged as a key area of interest. Gut microbiota-derived metabolites are believed to traverse the blood–brain barrier, potentially supporting the immunosuppressive microenvironment characteristic of GBM and modulating the phenotype of tumor-associated macrophages, which play a crucial role in tumor progression (130–132).

These interconnected findings across various cancer types point toward a shared underlying concept: both local and distant microbial communities can shape cancer development and progression through metabolic and immune-related mechanisms.

## 6 The gut-health nexus: how diet, sleep, and physical activity modulate dysbiosis, obesity, and cancer risk

Lifestyle factors significantly influence the composition and function of the gut microbiome, and the resulting dysbiosis can initiate a cycle that promotes obesity and, ultimately, increases cancer risk. This cycle is fueled by the interplay between dysbiosis, obesity, and lifestyle factors, further exacerbating dysbiosis. We will analyze how these factors interact.

### 6.1 Diet

There is a growing body of research examining the connection between diet, gut microbiota composition/diversity, and their impact on obesity and cancer development. There are three gut microbiome enterotypes according to high abundances of specific bacterial groups in healthy individuals: *Bacteroides* (Enterotype 1); *Prevotella* (Enterotype 2); and *Ruminococcus* (Enterotype 3). It is possible these broad profiles are associated with different dietary patterns: for instance, individuals with a diet high in protein and animal fat show a higher abundance of *Bacteroides*, compared to individuals who consume diets high in carbohydrates, who have a higher ratio of *Prevotella* (133).

Several diets have been investigated with regards to their association with nutrient intake and cancer. One of the most studied diets in relation to health and disease is the Mediterranean diet (MD), although it is now accepted that the MD is not so much a dietary pattern as a way of life (134). The MD is high in fruit, vegetables, olive oil (with anacidity rate lower than 0.8%), wholegrains, moderate consumption of fish, red wine and dairy, and low intake of red meats (135). The high consumption of fruit and vegetables means the diet is high in micronutrients and antioxidants with anti-inflammatory properties providing protection to the cell membranes from free radicals, reduction in the proliferation of cancer cells, the prevention of damage to DNA, and reducing pro-inflammatory signaling (136). With one third of cancer mortality being linked to diet and the associated inflammation caused by certain foods, the MD with its naturally anti-inflammatory properties shows promise in reducing the risk of certain cancers. A large study by (137), for instance, found a weak association between anti-inflammatory diet risk of cancer, although oddly, no evidence for protection from CRC. Ricceri, Giraudo (138) found that women who followed the MD had a 50% less risk of developing endometrial cancer than those who either did not follow the MD or had a low adherence to it. Shively, Register (139) found that monkeys who consumed an MD over a prolonged period of time (31 months) had a shift in the microbiome in their mammary glands tenfold in comparison to those consuming a Western-style diet.

In contrast to the MD, the WD is associated with increased risk of a number of non-communicable diseases (NCD) including cancer, and a sharp increase in obesity. The WD is characterized by a high intake of processed foods, meats, refined sugars, sweets and caloric drinks (140). A diet that is high in processed foods and refined sugars is associated with high levels of inflammation and an increased risk of CRC (11), and the low intake of dietary fibre and the increased intake of sugar and fats in the WD is hypothesized as being the leading cause of dysbiosis, notably an increased number of *Bacterioides* spp. (141). The WD has also been found to have an impact on the diversity of the gut microbiota, with a study on immigrants in the US showing a replacement of *Prevotella*, which is responsible for breaking down plant fibre, by dominant strains from the *Bacteroides* genus (142). This shift characterizes the first enterotype described by Güven Gülhan, Nikerel (143), which is marked by high *Bacteroides* levels and is commonly observed in individuals following a long-term WD. Similarly, levels of *Fusobacterium nucleatum*, a bacterium linked with CRC, increased following a 2-week WD intervention that consisted of high-fat and low fibre (144). The dysbiosis brought about by the WD also causes the mucosal lining to become thinner and a low-grade persistent inflammation to occur (145).

The documented benefits of fruits and vegetables would suggest adopting a plant-based diet pattern such as a vegan/vegetarian diet would be the best option for a healthy gut microbiome in relation to health and disease. However, a meta-analysis (146) found there was no evidence to support a vegetarian diet in the prevention of cancer mortality compared to a non-vegetarian diet, despite the anti-inflammatory and anti-oxidative effects provided by fruits and vegetables against the development and progression of cancer. In contradiction to this, one study (147) found that Chinese people who consume four to five servings of fruit, vegetables and legumes daily had a reduced risk of cancer mortality, although this could be different for hormone dependent cancers. This is supported by a finding that the consumption of legumes and lentils was linked to a 49% reduction in the risk of cancer mortality (148).

Based on studies such as those above, some attempts have been made to change the gut microbiota with targeted dietary interventions. Wastyk, Fragiadakis (149) assessed the influence of two such dietary interventions in healthy adults, one a plant-based fibre diet, and the second a diet based on fermented foods. The plant-based fibre diet showed no change in alpha diversity but an increase in microbial proteins per gram of stool, possibly showing a change in microbial density due to the increased fibre consumption. Surprisingly, there was no change in the levels of SCFA such as butyrate, which had been reported in other studies. The participants who consumed the fermented food diet showed an increase in alpha diversity as well as a decrease in inflammatory proteins. In another study (6), the “Microbiome Enhancer Diet” (MBD) aimed to ensure more nutritional components reached the colon in order to influence the gut microbiome. The diet was centered around four key components: increased consumption of dietary fibre; increased resistant starch; larger food particle sizes; and as little processed food ingredients as possible. The findings reveled that when compared to the WD, participants on the MBD excreted significantly more calories in their feces meaning they absorbed a smaller percentage of the consumed energy on the MBD compared to the statistically significant (*p* < 0.0001) higher absorption rate seen in the participants on the WD. Notably, this difference was seen to occur in energy absorption without any changes in the individual’s energy expenditure, feelings of hunger or the amount of food consumed. This study suggests there is a possibility the MBD creates a gut environment where more calories pass through the digestive system unabsorbed, providing metabolic benefits without the need to eat less or experience increased hunger. The reduction in the calories absorbed appears to be as a result of how the gut microbiome interacts with food rather than through behavioral modifications. No change was observed in alpha-diversity, but beta-diversity highlighted a significant difference.

Attempts to modify the gut microbiome through dietary changes face several significant challenges due to the nature of existing microbial communities. The gut microbiome is usually well established in adults and is resilient to temporary changes, actively resisting modification (150). Long-term dietary patterns/ lifestyles of an individual create metabolic pathways and selective pressures that favor the growth of certain bacterial populations. Established microbial colonies are known to occupy niches in the gut. Thus, new beneficial microbes introduced through diet face competitive disadvantage against well-established populations that have optimized their environment over time. Research indicates that creating a change in the gut microbiome with diet requires a sustained intervention lasting a minimum of 6 months (151).

### 6.2 Physical activity

Dysbiosis and obesity can indirectly affect physical activity levels when obesity-related inflammation, fatigue, or discomfort reduce the individual’s motivation or ability to engage in physical activity. A sedentary lifestyle can further disrupt the gut microbiome, creating a vicious cycle of dysbiosis, reduced physical activity, and weight gain.

Physical activity is widely recognized as a key factor in promoting overall health, reducing the risk of chronic diseases such as obesity and cancer, and influencing gut microbiome composition. Research has demonstrated the connection between physical activity and increased diversity of the gut microbiota: athletes and individuals who partake in regular vigorous exercise exhibit a more diverse gut microbial population. The difference in diversity is not only seen in the number of species but in the types of species, with shifts in bacterial composition (152). Several studies (35, 36, 153) show that exercise has been associated with an increase in microbial richness and the proliferation of beneficial bacteria such as *Bifidobacterium*, *Akkermansia muciniphila*, and *Faecalibacterium prausnitzii*, which are known to have anti-inflammatory effects.

Physical activity has also been shown to increase levels of SCFAs, particularly butyrate, which supports gut barrier integrity (see Section 3.1). Exercise helps prevent the translocation of LPS – a key driver of chronic inflammation and metabolic dysregulation – from the gut into the bloodstream (154). The anti-inflammatory effects of physical activity have been shown to alter the composition of the gut microbiota, reducing circulating LPS, and decreasing the levels of IL-6 and TNF-*α*. However, not all exercise has the same outcomes with effects depending on intensity, duration, and type of physical activity. Aerobic exercises such as running and cycling have been shown to enhance microbial diversity more than resistance training. Further, a review by Clauss, Gérard (155) has shown that too much exercise can actually cause harm, and excessively high-intensity exercise can lead to an increased gut permeability, a decrease in gut mucus thickness, and dysbiosis. Physical activity is associated with a reduced risk of several types of cancer, including CRC, breast, and prostate cancer (156). There are a number of pathways by which this may come about, including reduction of inflammation and regulation of insulin and glucose metabolism. The cycle of diet-induced dysbiosis, hormonal and sleep disruption, and reduced physical activity creates a self-reinforcing loop that promotes obesity. Obesity, in turn, further exacerbates dysbiosis and the risk of certain cancers.

Physical activity and exercise have been shown to influences gut transit time and motility, which in turn affects microbial composition and function. Physical activity and exercise accelerates the transit time in the gastrointestinal tract, reducing the opportunity for pathogenic bacterial colonization and promoting the growth of beneficial bacteria that can adapt to this environment Studies have shown that moderate-intensity exercise enhances colonic motility and reduces transit time (157, 158). This altered motility affects substrate availability for different microbial populations, favoring the growth of specific bacterial communities that can thrive under these conditions. The enhanced gut motility also aids in the mechanical removal of potential pathogens, reducing their residence time in the gut.

Physical activity and exercise influence the stress response through effects on the hypothalamic–pituitary–adrenal (HPA) axis and the sympathetic nervous system. These neuroendocrine pathways influence gut physiology and microbial composition. Regular moderate exercise reduces chronic stress and cortisol levels (159), which have been associated with increased intestinal permeability and dysbiosis. In contrast, excessive or high-intensity exercise may induce acute stress responses that temporarily affect gut barrier function and microbial composition (160). The integrity of the intestinal barrier is crucial for preventing translocation of bacteria and bacterial products from the gut lumen into systemic circulation, and physical activity has been shown to enhance intestinal barrier function through multiple mechanisms. Moderate exercise upregulates the expression of tight junction proteins that maintain epithelial barrier integrity (161). Exercise also promotes the production of heat shock proteins (HSPs) and intestinal alkaline phosphatase (IAP), which protect against stress-induced damage to the intestinal epithelium and detoxify bacterial endotoxins (162). Improved barrier function prevents bacterial translocation and the subsequent inflammatory response, creating a more favorable environment for beneficial microbes.

While the focus has been on how exercise affects the gut microbiome, it’s important to acknowledge the bidirectional nature of this relationship. Evidence suggests that the gut microbiome may influence exercise performance and adaptations to training. For instance, microbially derived metabolites, particularly SCFAs, enhance energy harvesting, muscle function, and endurance capacity (88).

### 6.3 Sleep

Emerging research is revealing a complex relationship between gut microbiome composition and sleep patterns. Far from being independent biological processes as previously thought, gut dysbiosis and sleep demonstrate a bidirectional interaction that significantly impacts human health and metabolic function.

The gut microbiome plays an important role in the production of neurotransmitters (see Section 4.4), particularly serotonin and melatonin, which are fundamental to sleep regulation. Approximately 95% of the body’s serotonin and a significant portion of melatonin are produced in the gut, highlighting the microbiome’s direct neurochemical influence (103). Disruptions in microbial composition can alter these critical neurotransmitter pathways, potentially compromising sleep quality and circadian rhythms. An imbalance in the microbial population can trigger increased production of pro-inflammatory cytokines, which disrupt normal sleep architecture. These inflammatory markers activate neural pathways that interfere with sleep onset, maintenance, and overall quality (163). The circadian rhythm governs not only sleep–wake cycles but also microbial populations, with disrupted sleep leading to shifts in microbial diversity, potentially reducing beneficial bacterial populations and promoting inflammatory microorganisms (164). Poor sleep has been shown to alter the gut microbiome composition: a decrease in sleep duration and quality associated with gut dysbiosis creates a negative feedback loop with sleep deprivation causing an increase in appetite hormones, cravings for calorie-dense foods, reduced metabolic efficiency and decreased physical activity (165). These factors contribute to weight gain and obesity, further exacerbating gut microbiome imbalances. Disrupted sleep patterns have been linked to cancer risk, with growing evidence suggesting that the gut microbiome may also play a role in this relationship. Sleep deprivation and disruptions to the circadian rhythm, such as those seen in shift can lead to gut dysbiosis with a reduction in abundance of species such as *Bifidobacterium* leading to inflammation (166). Furthermore, a lack of quality sleep is linked to increased levels of pro-inflammatory which create a pro-tumorigenic environment and accelerates cancer progression (167).

## 7 Stress, anxiety, and the gut: bidirectional interactions

The connection between psychological states and gut function is a strong example of mind–body interaction. The gut-brain axis, a well-established network linking the central and enteric nervous systems, acts as the key pathway through which mental states impact digestive processes and vice versa. Understanding these interactions offers insights for developing integrated approaches to managing both psychological and gastrointestinal disorders. The gut-brain axis encompasses multiple pathways that enable bidirectional communication between the central nervous system and the gastrointestinal tract. For example, the vagus nerve is the primary component of the parasympathetic nervous system innervating the gut (the “second brain”), and 80–90% of its component nerve fibres are afferent carriers of information from the gut to the brain. The hypothalamic–pituitary–adrenal axis, a neuroendocrine system that controls stress responses and influences gut function through the release of corticotropin-releasing factor (CRF), adrenocorticotropic hormone (ACTH), and cortisol, and through microbial signaling that can affect brain function, including neurotransmitters such as serotonin, gamma-aminobutyric acid (GABA), and SCFAs.

Acute stress speeds up the transit time in the colon whilst delaying gastric emptying, a pattern mediated primarily by CRF. In animal models, CRF administration mimics stress-induced alterations in gut motility, while CRF antagonists block these effects (168). In humans, these changes to motility are thought to be an evolutionarily adaptation, preparing the organism for “fight or flight” by diverting resources away from digestion. However, chronic activation of this system can lead to motility issues. Stress and anxiety contribute to “leaky gut,” through alterations to the tight junction proteins and disruption of the intestinal mucus layer (169). Transferring fecal microbiota from depressed patients to microbiota-depleted rats showed an increase in intestinal permeability and depressive-like behaviors (170). These stress and anxiety-induced changes in turn induce significant alterations in gut microbial composition, with a reduction in microbial diversity, a decrease in beneficial species, an increase in pathogenic species, and an alteration in metabolite production. These changes have been noted as taking place within a relatively short period of time after the stressor has occurred in mice (171). Diets that have a particularly high intake of refined sugar, significantly influence the gut microbiome and, consequently, anxiety levels. This has been demonstrated in Western-style diets, with their high refined sugars and associated selective promotion of pathogenic bacteria (e.g., certain *Clostridia*). High-sugar diets decrease the abundance of bacteria that produce butyrate and other SCFAs, which are crucial for maintaining intestinal barrier integrity and have anti-inflammatory properties. Lower SCFA levels are associated with increased gut permeability and systemic inflammation. a reduction in beneficial SCFA-producing bacteria, decreased microbial diversity, and increased intestinal permeability (172, 173). Magnusson, Hauck (174) demonstrated that high-sugar diets promoted the growth of Proteobacteria while reducing beneficial Bacteroidetes, leading to intestinal dysbiosis. This dysbiosis was correlated with increased anxiety-like behaviors in rodent models (175). Stress and depression have been seen to reduce physical activity through pathways such low energy levels, reduced pleasure in activities, and poor sleep pattern (27, 176). Studies have consistently shown that individuals with depression engage in significantly less physical activity than non-depressed controls (177). One meta-analysis (178) found that people with depression are 50–60% more likely to be physically inactive compared to the general population.

The interplay between depression, physical activity, and gut health appears to create a self-perpetuating cycle in which depression reduces physical activity, leading to alterations in gut microbiome composition and function. This gut dysbiosis contributes to intestinal inflammation and increased permeability, allowing inflammatory mediators and bacterial translocation to influence brain function. The resulting neuroinflammation exacerbates depressive symptoms, further deepening the cycle by reducing physical activity even more. Cancer diagnosis has a huge psychological impact on an individual. Lee, Nam (179) found that psychiatric disorders were common in patients with cancer and patients with cancer and a newly diagnosed psychiatric disorder had a higher mortality rate. This supports (180) who highlighted that between 30 and 60% of cancer patients had a psychiatric disorder such as extreme stress, depression, anxiety and insomnia. As of yet there are no studies showing how the mechanistic role of the gut microbiome influences stress, depression and anxiety in relation to cancer.

## 8 The promising potential of microbial reprogramming

The reprogramming of the gut microbiome through therapeutic interventions has recently been shown as a promising tool to address a number of diseases. This includes the use of probiotics to restore microbial balance and fecal microbiota transplant (FMT) which has been used to completely replace a recipient’s gut microbiome.

### 8.1 Microbial reprogramming via probiotics

Probiotics – live microorganisms that, when administered in adequate amounts, confer a health benefit on the host – are another avenue for potentially reprogramming the gut microbiome (181). The idea is that introducing beneficial bacteria can help to restore balance to the gut, although the effects can be variable and depend on the specific strains used, the individual’s existing microbiome, and other factors such as diet. Probiotics can be used to modulate the gut microbiota by releasing SCFA such as butyric or acetic acid, which can help restore balance to a microbiome in dysbiosis and help to improve intestinal permeability and gut barrier function (182). Probiotics such as *Lactobacillus* and *Bifidobacterium* can control obesity through regulating the functions of the hosts own gut microbiome (183). It has been shown in animal models that probiotics have the potential to produce anticancer effects through the regulation of the gut microbiota and thus achieve immune modulation to reduce chronic inflammation by modulating both Toll-like receptors (TLRs) and G-protein coupled receptors (GPRs), lowering intestinal pH, and inhibiting enzymes that produce carcinogens (184, 185). However, it is worth noting that the effects and results from the use of probiotics are strain-specific and so may vary from individual to individual (186).

### 8.2 Microbial reprogramming via fecal microbiota transplant

FMT is being explored as a strategy to reprogram the gut microbiome in the context of obesity and related metabolic disorders. It represents one of the most direct interventions for microbiome reprogramming currently available (187). While traditional dietary and probiotic approaches offer incremental changes to the gut ecosystem, FMT provides a complete microbial community transfer, potentially offering more rapid and comprehensive microbiome reprogramming. Although the use of fecal matter is not new, with ancient Chinese medicine using ‘yellow soup’ as a treatment for diarrhea, it was not until the early 21^st^ century that FMT has gained recognition as a viable tool for treating gut dysbiosis and its associated diseases. A start has been made to use FMT to treat a number of diseases such as inflammatory bowel disease (IBD). A systematic review by Paramsothy, Paramsothy (188) found 41 studies with overall clinical remission rates of 36% for ulcerative colitis, 50.5% for Crohn’s disease, and 21.5% for pouchitis following FMT. Research investigating the gut-brain axis has also prompted exploration of FMT for neuropsychiatric conditions. Preliminary studies in autism spectrum disorder (ASD) have shown promising results: Kang, Adams (189) reported improved gastrointestinal and behavioral symptoms in children with ASD following microbiota transfer therapy, with benefits persisting two years post-treatment. In relation to obesity, a meta-analysis looking at 10 studies for a total of 334 participants showed that individuals who received FMT showed a negative association with calorie intake, fasting glucose levels, and total cholesterol (190). Zhang, Zuo (191) showed minor weight loss in obese patients who received FMT and an increase in *Bacteroides* in the mucosal microbiome in the colon, but FMT appeared to have less influence over changing the composition of the microbiota of the small intestine.

While FMT may be a direct and potentially powerful intervention, there are still subtilties in its use. For instance, two studies (192, 193) showed no difference in fat mass, lean mass, or metabolic parameters in individuals who had received FMT after 12 weeks. In addition, a study comparing young mice (3 months) vs. older mice (24 months) found that key species were transferred between the mice despite the differences in age, with the older mice developing an increased intestinal barrier when receiving FMT from the young mice (194). However, when the young mice received FMT from the older mice the inflammatory cytokine levels of IL- and TNF- became elevated to match that of the older mice donors. This study shows the influence of the FMT on the recipient and why the screening and history of the donor is vital before considering any transplant. Despite these caveats, FMT is firmly established for treating recurrent *C. difficile* infection (195), and its potential extends to numerous conditions with emerging evidence supporting applications in inflammatory, metabolic, and neuropsychiatric disorders. In the context of cancer, FMT has been seen as a way to improve the efficacy of immunotherapy and a reduction in associated toxic events. Species such as *Bifidobacterium fragilis* were found to have anti-cancer properties (196). As the interplay between gut microbiota, health and disease becomes more understood, FMT will likely play an increasingly important role in microbiome reprogramming across a number of diseases.

## 9 Conclusion

Lifestyle plays a role in the development of both obesity and cancer, with factors such as diet, physical activity, and sleep known to influence disease risk and progression. This review proposes a mechanistic model that places the gut microbiome at the intersection of these lifestyle factors and disease processes. Dysbiosis of the gut microbiota has been strongly associated not only with obesity but also with the development and progression of cancers such as colorectal and breast cancers where mechanistic pathways are best characterized, and there is emerging but compelling evidence for pancreatic and other malignancies (15, 17, 128). Microbial species have been implicated in promoting obesity through mechanisms such as enhanced energy harvest from the diet, increased fat storage, modulation of appetite regulation, disruption of circadian rhythms, and the promotion of low-grade chronic inflammation (5, 38, 51, 53).

The review demonstrates a cycle of lifestyle-induced dysbiosis that promotes obesity, which further disrupts the microbial balance. These pathways offer promising targets for intervention, as shown by initiatives like the BE GONE trial, which targeted microbiome modulation in obese patients with history of colorectal neoplasia to mitigate cancer risk. At the end of the 16 weeks trial the study reported an increased alpha diversity in the participants who consumed the prebiotics foods (197).

Studies have highlighted how physical activity can be an influential microbiome modulator with implications for cancer prevention. Beyond its established benefits for energy balance and systemic inflammation, exercise creates distinct alterations in gut microbial ecosystems through physiological mechanisms (198, 199). Regular physical activity enhances microbiome diversity, a key indicator of gut health whilst simultaneously enriching beneficial bacterial species associated with improved metabolic health and immune function (200, 201). Exercise-induced changes in transit time, mucosal immunity, and bile acid metabolism collectively help to shape the intestinal environment, promoting microbial profiles linked to a reduced cancer risk (202, 203).

While many studies report associations between physical activity, diet, and microbial diversity, only a few interventional trials have demonstrated causative links of lifestyle factors to cancer outcomes. One such trial was conducted by Wastyk, Fragiadakis (149), who found that diet significantly influenced the gut microbiome, which in turn affects immune function. In a 17-week randomized study involving healthy adults, the researchers compared the effects of two dietary interventions, a high-fibre diet, and a diet focused on fermented foods. The fermented food diet led to a steady increase in microbiota diversity and a reduction in inflammation. These findings suggest that fermented foods may be particularly effective in improving microbiome health and lowering inflammation. A recent meta-analysis showed consumption of fermented dairy products such as yogurt was significantly linked with a decreased risk of cancers such as bladder, CRC and esophageal. Kefir, a fermented milk, has also been shown to have potential in the prevention and treatment of cancer through its anti-bacterial and anti-inflammatory properties (149, 204, 205).

**Figure 3:**
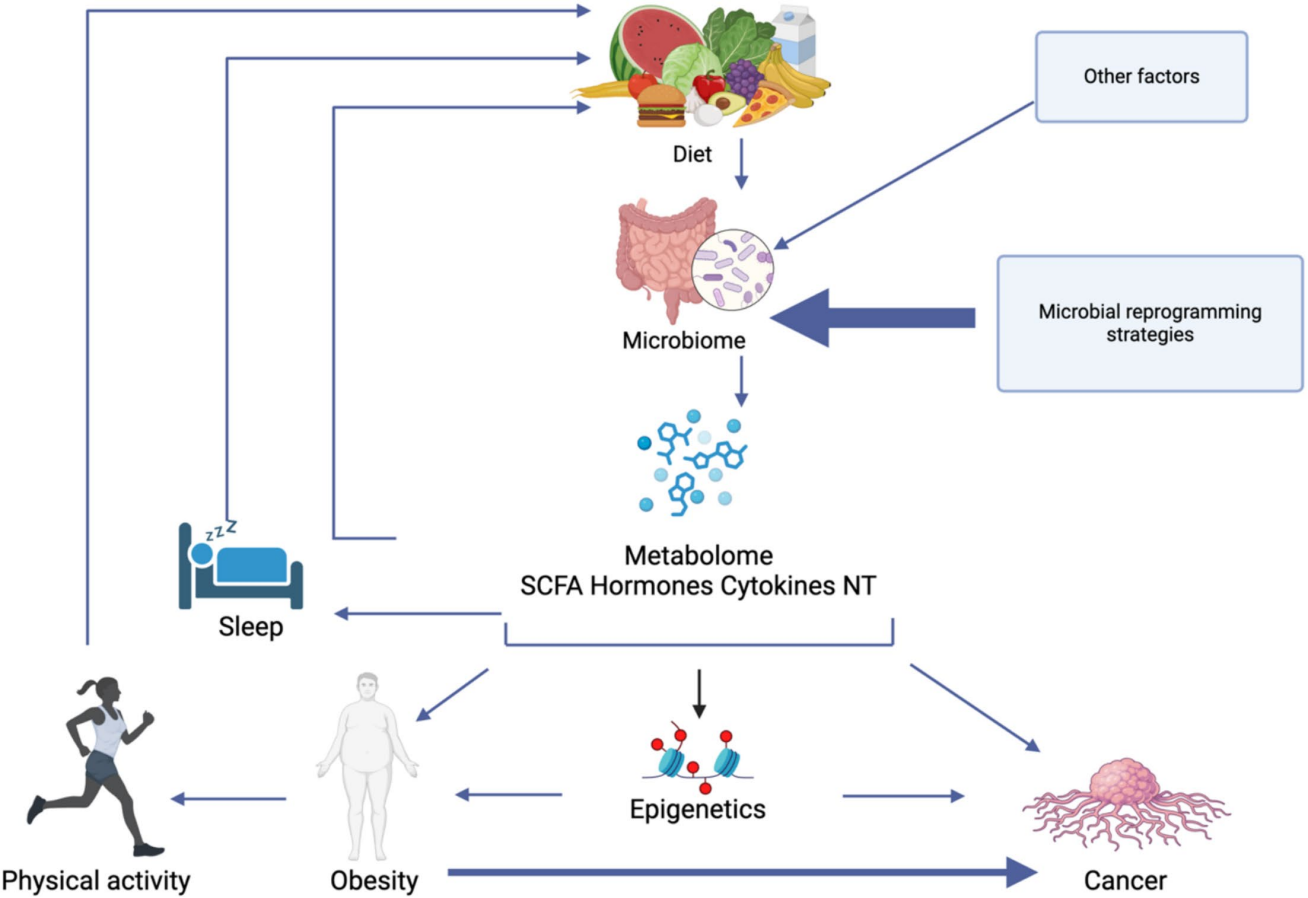
A model of the complex interplay between lifestyle factors, gut microbiome composition, and metabolic outcomes that influence obesity and cancer development. Diet directly modulates the gut microbiome, which in turn produces various metabolites including short-chain fatty acids (SCFAs), hormones, cytokines, and neurotransmitters (NT). These metabolites mediate numerous physiological effects, including epigenetic modifications that can influence both obesity and cancer pathways. Sleep quality and physical activity both influence and are influenced by the microbiome-metabolite axis. Created in BioRender.

Significant gaps remain in understanding the precise mechanisms and how they interact with modifiable lifestyle factors. Future research would benefit from studies that can establish causality rather than correlation, examining how physical activity, dietary patterns, and sleep quality can modulate microbiome composition over time. Additional to this is the importance of addressing individual variability by exploring how genetics, sex, age, and the individual’s environment can influence the microbiome in relation to lifestyle interventions (Figure 3).
